# Translation and cultural adaptation of Glasgow Antipsychotic Side-effects Scale (GASS) in Arabic

**DOI:** 10.1371/journal.pone.0201225

**Published:** 2018-08-23

**Authors:** Yazed AlRuthia, Hadeel Alkofide, Fahad Dakheel Alosaimi, Hisham Alkadi, Albandari Alnasser, Aliah Aldahash, Arwa Basalamah, Maryam Alarfaj

**Affiliations:** 1 Department of Clinical Pharmacy, College of Pharmacy, King Saud University, Riyadh, Saudi Arabia; 2 Department of Psychiatry, College of Medicine, King Saud University, Riyadh, Saudi Arabia; 3 Department of Teacher Training, Arabic Linguistics Institute, King Saud University, Riyadh, Saudi Arabia; Peking University, Institute of Mental Health, CHINA

## Abstract

**Background:**

In view of a constant increase in the number of patients treated with antipsychotic medications, the problem of nonadherence to the prescribed treatment becomes particularly relevant. Since one of the major contributors to the nonadherence is the presence of side effects of the drugs being used, the availability of tools for assessment of adverse reactions is of great importance.

**Objective:**

The objective of the present work was to develop an Arabic language version of the Glasgow Antipsychotic Side-effect Scale (GASS).

**Methods:**

After confirming the accuracy of translation, the questionnaire was given to 100 patients in two psychiatric centers in Saudi Arabia.

**Results:**

The Cronbach’s alpha (0.793) indicated a good reliability of the survey. The mean GASS score was 19.09, indicating absent or mild side effects of antipsychotics, but 46% of patients experienced moderate, and 25% experienced severe side effects. An analysis of the correlation between patients’ characteristics and side effects revealed the presence of a positive relationship between the side effects and health literacy.

**Conclusions:**

It is expected that the Arabic-GASS will benefit Arabic-speaking psychiatric patients by helping them to express their concerns about side effects of antipsychotics. The collected results also document the importance of patients’ health literacy in achieving high-quality healthcare.

## Introduction

Recent decades have witnessed a dramatic increase in the number of prescriptions dispensed for psychotropic medications. A two-fold increase was noted in the United States from 1995 to 2005 and a more than four-fold increase was documented in Italy from 2000 to 2011 [1,2]. Similar patterns have been seen worldwide [3–5]. In Saudi Arabia, antipsychotics were the most frequently prescribed psychotropic medications [6]. However, medication adherence among psychiatric patients is poor [7, 8], and was reported to range from 18 to 80% [9–11]. The variability in the reported values may reflect inherent limitations of the different methods that were used to assess adherence in different studies [9]. In this regard, the most stringent approach based on serum levels of the prescribed drug revealed that almost 60% of schizophrenic patients are not adherent or poorly adherent to the prescribed treatment [12]. The analysis of the nonadherence patterns is further complicated by the fact that the behavior of individual patients may change over time; this makes the fraction of patients characterized by a consistent good adherence even smaller [13].

Nonadherence to antipsychotics is one of the major risk factors for poor treatment outcomes [14]. Lack of patient adherence to prescribed treatment increases the probability of relapse and lowers the odds of remission [7, 15]. It also constitutes a significant predictor of hospitalization among patients taking antipsychotics [16], and prognosticates an increase in the annual cost of the disease and the need for external services [17]. It was estimated that the annual cost of rehospitalization resulting from nonadherence to antipsychotic medications was between $1.4 and $1.8 billion in the United States alone, and this value reflects only patients covered by the Medicaid program [18]. Therefore, it becomes essential to maximize patients’ adherence to their prescribed medications.

It must be recognized that a high percentage of discontinuation cases of antipsychotics involved decisions by patients against their physicians’ recommendations [10, 19]. Approximately half of these instances are caused by the decisions of the patients themselves [20, 21]. An important factor leading to this unwanted behavior is a negative attitude of patients towards medications [15]. Not surprisingly, medication nonadherence is strongly associated with patient-reported side effects [22–24], and the impact of side effects was shown to be a potent independent predictor of medication omission [25]. The side effects that are most often correlated with increased nonadherence are cognitive dysfunction and distress over the weight gain [22, 26]. It was also pointed out that the fear of side effects, as opposed to the actual experience, may increase the risk of nonadherence [27].

Several scales designed to rate the side effects of antipsychotic drugs were being proposed since 1970 [28–32]. Their limitations included the necessity to be administered by a physician [28, 30], or being lengthy and time-consuming [29, 32]. Also, difficulties were encountered by the patients in comprehending the terminology used in self-report questionnaires [31, 33]. In order to overcome these obstacles, Waddell and Taylor, developed an improved self-report rating scale to evaluate side effects of second-generation antipsychotic drugs, the Glasgow Antipsychotic Side-effect Scale (GASS) [34]. Unlike the older tools which focused on selected side effects, mostly movement disorders, GASS addresses sedation, central nervous system, cardiovascular, extrapyramidal, anticholergenic, gastrointestinal, genitourinary, and prolactinemic side effects. It also asks about indicators of diabetes and the presence of weight gain. This scale contains 22 questions in simple English language, it requires 5 minutes to complete, and can be easily understood by the patients [34]. Besides its brevity and comprehensiveness, the GASS is the only self-report antipsychotics’ side effects scale in which mental health consumers and experts were involved in its items generation compared to Antipsychotic Non-Neurological Side-Effects Rating Scale (ANNSERS), Liverpool University Neuroleptic Side Effect Rating Scale (LUNSERS), Maryland Psychiatric Research Center Scale (MPRC), Nursing Extra Pyramidal Symptoms Assessment Scale (NEPSAS), Prince Henry Hospital Akathisia Rating Scale (PHHARS), and Yale Extrapyramidal Symptom Scale (YESS) [35]. Furthermore, the GASS is a generic scale that can be used for all antipsychotics and not an antipsychotic specific scale such as the GASS for clozapine [36–39].

It has been shown that patients’ health literacy can affect their behavior and clinical outcomes [40, 41]. Health literacy is known to have an impact on medication adherence [42, 43]. Lack or limited health literacy is associated with increased unintentional nonadherence; however, it is not implicated in intentional nonadherence [44]. A more complex pattern of behavior was also observed: limited or low health literacy was associated with unintentional nonadherence, while adequate or high health literacy was associated with intentional nonadherence. The most adherent group is constituted by patients with moderate health literacy [42, 45]. However, little information is available on the correlation of health literacy and reporting of medications’ side effects.

The aim of this study was to generate an Arabic version of GASS and test its performance in Arabic speaking population. Availability of GASS in the Arabic language would facilitate the detection and reporting of side effects of antipsychotics in psychiatry clinics in the Middle East countries, in which Arabic is spoken by the vast majority of the population. The relationship between health literacy and reporting of side effects was also explored.

## Materials and methods

### Study design and data source

The permission for validating and using the GASS questionnaire was granted by Dr. Linda Waddell (Glasgow, UK) [34]. The forward translation of GASS was performed by an English linguist whose native language is Arabic. Then, an English native speaking healthcare provider with high Arabic proficiency, translated the Arabic version of GASS back to English. The English and Arabic versions of GASS did not show any significant differences. The Arabic translation of GASS was then reviewed by a psychiatrist and a clinical pharmacist to ensure face and content validity. Thereafter, the final Arabic version of GASS (Arabic-GASS) was reviewed and, after addressing all comments, approved by all members of the research team. The study adhered to the methodological guidelines for translation, adaptation, and validation of self-reported screening instruments [46]. The study was approved by the Institutional Review Boards of the Psychiatry Clinic at the King Khalid University Hospital in Riyadh and of the Mental Health Hospital in Hafar Al-Batin.

### Study population

The participants of this cross-sectional study were recruited between June 7, 2016, and August 3, 2017, among the patients of the Psychiatry Clinic at the King Khalid University Hospital in Riyadh and of the Mental Health Hospital in Hafar Al-Batin, Saudi Arabia. Convenience sampling method was used. Inclusion criteria were: age 18 years or older, being on antipsychotic drug(s) for 3 months or more, ability to read and understand Arabic language or availability of help from a family member, and consent to participate in the study. Patients’ electronic medical records were reviewed to verify that they do receive the medications. Patients who were not on antipsychotics were excluded.

Subjects were interviewed by two pharmacy interns to provide socioeconomic characteristics of the patient population (e.g., age, gender, and education). The interviews took place in private rooms to ensure the privacy of the participants. Illiterate patients were helped by their family members. Information about antipsychotics’ dosage, dosage forms, duration of illness, comorbidities, and other prescription drugs were collected from the patients’ electronic medical records. The Charlson comorbidity index (CCI) was used to measure comorbidities [47]. Health literacy was assessed using the Arabic version of the Single Item Literacy Screener (SILS) [48–50]. This tool consists of a single question that assesses the respondent’s ability to read and comprehend educational information and instructions about different medical conditions and medications without anyone’s help [48].

### Statistical analysis

To safeguard the confidentiality, all data were coded, and no patient identifiers were collected. Student’s t-test and chi-square test were used for descriptive statistics. The association between the patients’ GASS scores and their medical and socioeconomic characteristics was examined using Pearson correlation coefficient. Factor analysis with varimax rotation was performed to explore the structure of the Arabic-GASS. Cronbach’s alpha method was used to assess the internal consistency of the Arabic-GASS. Statistical significance was defined by p<0.05. All analyses were conducted using the SAS statistical software (version 9.2, SAS Institute Inc., Cary, NC, USA).

## Results

**Fig 1:**
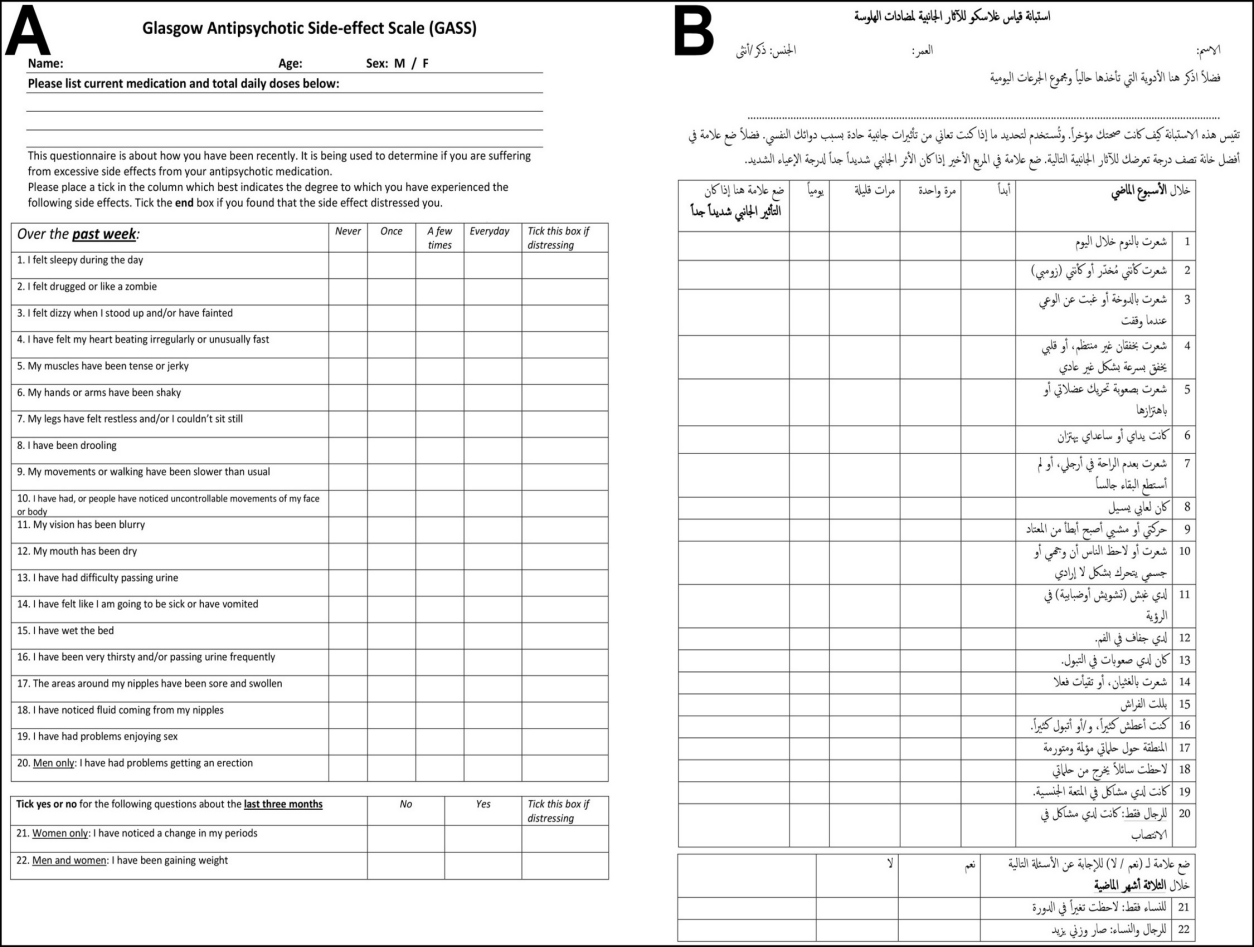
Comparison of the original and the translated questionnaire. A, original English language version of the GASS; B, the same document in Arabic (Arabic-GASS). Republished from [Waddell L, Taylor M. A new self-rating scale for detecting atypical or second-generation antipsychotic side effects. J Psychopharmacol. 2008;22: 238–243] under a CC BY license, with permission from [Waddell L], original copyright [2007].

The original and the Arabic language versions of the GASS are shown in Fig 1. The organization of the questions and possible answers are the same in both versions of the questionnaire. Importantly, bilingual or fluent in both languages healthcare professionals ensured that the translation of the Arabic version back into English yields wording almost identical to the original.

**Fig 2:**
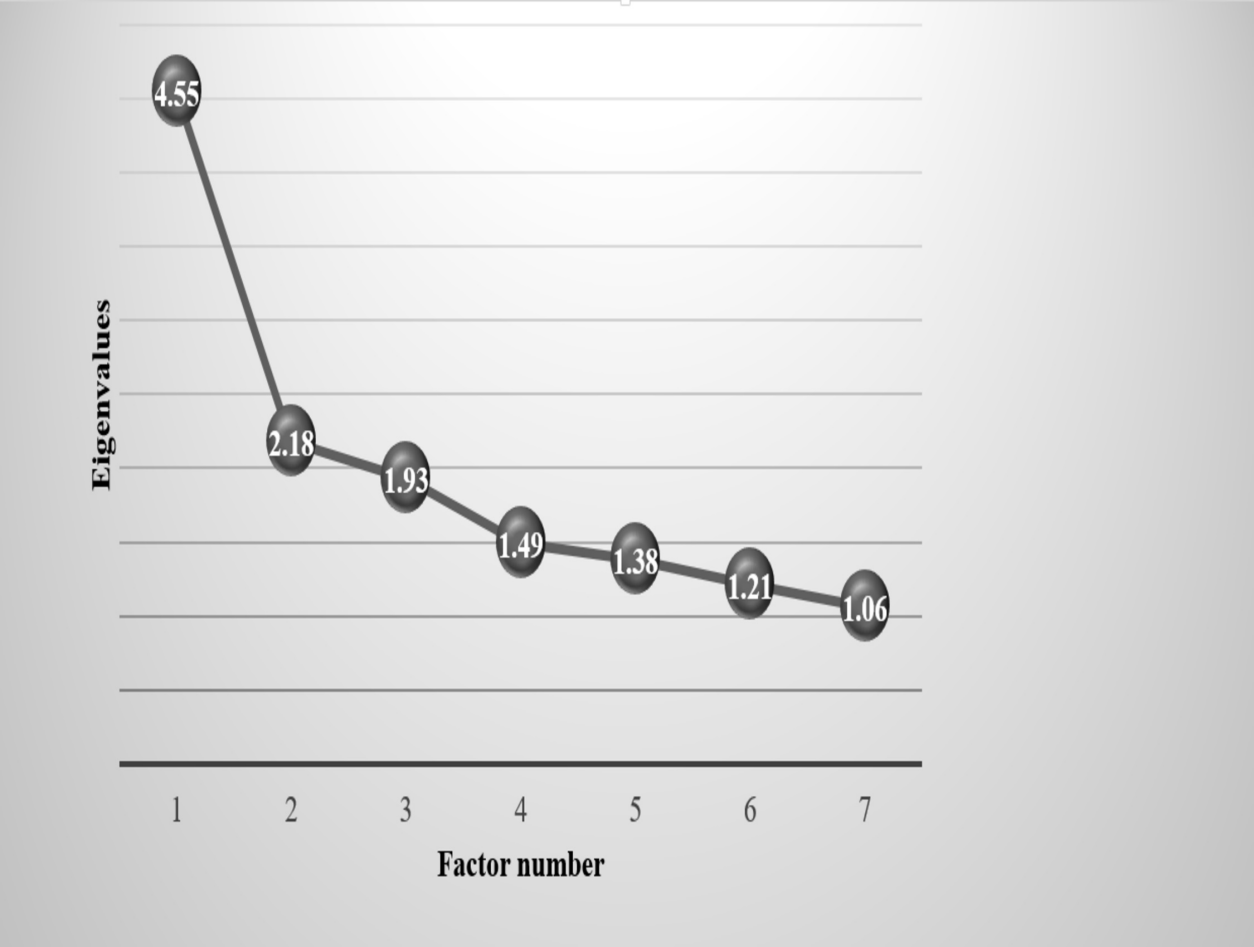
Scree plot of the number of factors that can be extracted from the Arabic version of GASS and their eigenvalues.

The questionnaire was presented to 100 patients being treated with antipsychotics. The participants did not report problems in answering the questions included in the form. Seven factors were extracted from the Arabic version of GASS using an eigenvalue cutoff point of ≥1 as shown in Fig 2. The highest loading of each item of the Arabic version of GASS is presented in Table 1. These factors included the following side effects: (1) orthostatic hypotension like symptoms (fainting, dizziness, irregular heartbeats, tremulousness, and blurry vision), (2) narcolepsy like symptoms (sleepiness, zombie-like state, slow-paced movement, and weight gain), (3) extrapyramidal and anticholinergic side effects (restless legs, uncontrollable movement, difficulty passing urine, and feeling thirsty), (4) sexual dysfunction side effects (problems enjoying sex and getting an erection for men), (5) cholinergic side effects (drooling, nausea, and vomiting), (6) hyperprolactinemia like symptoms (swollen and sore nipples, nipple discharge, and change in menstrual periods), and (7) abnormal muscle tone (jerky or tense muscles and involuntary urination). The value of Cronbach’s alpha was 0.793.

**Table 1:** Extracted factors from the Arabic version of Glasgow Antipsychotic Side-effect Scale (GASS).

Item	Factors							Communalities (h2)
	(1)	(2)	(3)	(4)	(5)	(6)	(7)	
**1. I felt sleepy during the day**		**0.745**						**0.636**
**2. I felt drugged or like a zombie**		**0.815**						**0.705**
**3. I felt dizzy when I stood up and/or have fainted**	**0.602**							**0.539**
**4. I have felt my heart beating irregularly or unusually fast**	**0.631**							**0.648**
**5. My muscles have been tense or jerky**							**0.613**	**0.623**
**6. My hands or arms have been shaky**	**0.656**							**0.694**
**7. My legs have felt restless and/or I couldn’t sit still**			**0.409**					**0.602**
**8. I have been drooling**					**0.796**			**0.759**
**9. My movements or walking have been slower than usual**		**0.648**						**0.656**
**10. I have had, or people have noticed uncontrollable movements of my face or body**			**0.654**					**0.508**
**11. My vision has been blurry**	**0.725**							**0.575**
**12. My mouth has been dry**			**0.484**					**0.444**
**13. I have had difficulty passing urine**			**0.803**					**0.705**
**14. I have felt like I am going to be sick or have vomited**					**0.670**			**0.717**
**15. I have wet the bed**							**0.758**	**0.721**
**16. I have been very thirsty and/or passing urine frequently**			**0.296**					**0.565**
**17. The areas around my nipples have been sore and swollen**						**0.293**		**0.4619**
**18. I have noticed fluid coming from my nipples**						**0.323**		**0.627**
**19. I have had problems enjoying sex**				**0.821**				**0.751**
**20. For men only: I have had problems getting an erection**				**0.643**				**0.811**
**21. For women only: I have noticed a change in my periods**						**0.778**		**0.664**
**22. I have been gaining weight**		**0.363**						**0.407**

The characteristics of the patients are shown in Table 2. They were mostly female (72%), young or middle-aged (less than 45 years of age, 75%), and approximately half of them (48%) were married. Although 64% had education at high school level or higher, only 16% were considered to have a good health literacy. Approximately three-quarters of patients were unemployed, and 43% of them lived in families with monthly income of less than $800. In most cases, patients were diagnosed with schizophrenia, a mental disease that lasted for more than 10 years, and the pharmacological treatment involved a single type of medication. A vast majority, 88%, of patients had a Charlson Comorbidity Index score of one or less. Some patients were on both first- and second-generation antipsychotics. The antipsychotic medications used by the patients are listed in Table 3.

**Table 2:** Baseline characteristics of psychiatric patients (N = 100) on antipsychotics.

Characteristic	Frequency N
**Sex**	
Male	28
Female	72
**Age (years)**	
18–24	8
25–34	27
35–44	40
45–54	19
55–64	4
≥65	2
**Marital status**	
Single	52
Married	48
**Education**	
Illiterate (unable to read or write)	4
Elementary school (1–6 years)	17
Intermediate school (7–9 years)	15
High school (10–12 years)	25
Some college or college degree (13–16 years)	33
Postgraduate degree (≥ 17 years)	6
**Health literacy**	
Marginal/limited	84
Good	16
**Employment**	
Unemployed	72
Employed	28
**Monthly income ($)**	
<800	43
800–1,600	20
1,600–2,666	19
2,666–4,000	10
4,000–5,333	7
5,333–6,666	1
**Mental illness**	
Depression	9
Bipolar disorder	37
Schizophrenia	54
**Duration of illness (years)**	
≤1	8
2–5	16
6–10	15
>10	61
**Number of prescription medications**	
1	58
2–4	31
5–7	9
≥ 8	2
**Charlson Comorbidity Index score**	
≤1	88
2	8
≥3	4

**Table 3:** Antipsychotic drugs used by the patients (n = 100).

Antipsychotic	Frequency n
Quetiapine	29
Risperidone	26
Olanzapine	20
Clozapine	17
Aripiprazole	13
Haloperidol	6
Paliperidone	6
Zuclopenthixol	3
Trifluoperazine	2
Sulpiride	1
Chlorpromazine	1

Table 4 illustrates the actual results of GASS questionnaire submitted by the 100 patients. The most frequent side effects of antipsychotic medications included sedation (sleepiness and feeling drugged), cardiovascular events (dizziness upon standing or fainting), extrapyramidal (drooling, impairment of movement), anticholinergic (dry mouth), and, in women, prolactinemic (change in menstruation pattern) effects. Weight gain was frequently reported, as well. The average GASS score in this population was 19.09±10.55, a number close to the upper limit of the “absent/mild side effects” range (Waddell and Taylor, 2008). However, when data for individual patients are considered, (Table 5), it becomes apparent that 46% of them experienced moderate, and 25% experienced severe side effects.

**Table 4:** Glasgow Antipsychotic Side-effect Scale (GASS) scores of the patients (n = 100).

Item	Frequency n	Mean ± SD
1. I felt sleepy during the day.	100	1.65±1.26
2. I felt drugged or like a zombie.	100	1.48±1.21
3. I felt dizzy when I stood up and/or have fainted.	100	1.04±1.17
4. I have felt my heart beating irregularly or unusually fast	100	0.80±1.10
5. My muscles have been tense or jerky.	100	0.86±1.21
6. My hands or arms have been shaky.	100	0.84±1.14
7. My legs have felt restless and/or I couldn’t sit still.	100	0.68±1.07
8. I have been drooling.	100	1.12±1.28
9. My movements or walking have been slower than usual	100	1.32±1.36
10. I have had, or people have noticed uncontrollable movements of my face or body	100	0.70±1.07
11. My vision has been blurry.	100	0.84±1.16
12. My mouth has been dry.	100	1.18±1.25
13. I have had difficulty passing urine.	100	0.40±0.94
14. I have felt like I am going to be sick or have vomited.	100	0.72±1.04
15. I have wet the bed.	100	0.38±0.86
16. I have been very thirsty and/or passing urine frequently.	100	0.32±1.32
17. The areas around my nipples have been sore and swollen	100	0.37±0.88
18. I have noticed fluid coming from my nipples.	100	0.13±0.58
19. I have had problems enjoying sex.	100	0.30±0.79
20. For men only: I have had problems getting an erection.	28	0.32±0.86
21. For women only: I have noticed a change in my periods.	72	1.05±1.44
22. I have been gaining weight.	100	1.53±1.51
**Total score**	100	19.03±10.55

**Table 5:** Severity of side effects of antipsychotics based on the GASS scores.

Severity class	Frequency n
Absent/Mild (0–12)	29
Moderate (13‐26)	46
Severe (>26)	25

An analysis of the correlation between patients’ characteristics and side effects of antipsychotic medications as defined by the GASS score was also performed and is shown in Table 6. The only statistically significant relationship found was the positive correlation between the presence of side effects and health literacy (r = 0.313, p = 0.001). The remaining patients’ characteristics, including the number of prescribed medications and the duration of illness, were not significantly associated with the GASS score.

**Table 6:** The correlation between GASS score and patients’ baseline characteristics.

Characteristic	Pearson Correlation Coefficient (r)	*P*-value
Age	0.025	0.803
Sex	-0.025	0.804
Education	-0.085	0.401
Employment	-0.0124	0.903
Health literacy	0.313	0.001*
Number of prescription medications	0.044	0.658
Charlson Comorbidity Index	-0.064	0.525
Income	-0.055	0.587
Marital status	0.078	0.435
Duration of illness	-0.001	0.987

*P<0.05

## Discussion

This study presents the first translation of the Glasgow Antipsychotic Side Effects Scale (GASS) to Arabic. Arabic is the sixth most frequently used language; it is estimated to be the native tongue for 420 million people worldwide. So far, no self-report assessment tool of antipsychotics’ side effects exists in Arabic. With the development of Arabic-GASS, this sizable population will have access to a simple and dependable tool for evaluation of antipsychotic medications’ side effects.

Recognizing and managing these side effects can improve the clinical outcomes and patient satisfaction. It will help to conduct systematic and open discussions between clinicians and patients and engage patients in decision making. Arabic-GASS will also be advantageous in medical research focused on the Middle East population and will promote better understating of the safety of antipsychotic drugs in Arab-speaking countries.

Arabic version of GASS demonstrated good reliability as indicated by Cronbach’s alpha coefficient of 0.793 [51]. Although the original report of GASS did not evaluate this parameter [34], recent modifications of this scale reported similar values, 0.83 and 0.903 [36, 38]. Thus, the internal consistency of the questionnaire was not altered in the process of translation. Original GASS had a good test-retest reliability, with the kappa value of 0.72; however, only 17 patients participated in the retest [34]. Although for logistic reasons test-retest was not performed in the current study, it is unlikely that the kappa coefficient would be meaningfully different in Arabic-GASS. Of note, the average GASS score obtained in the present investigation is 34% higher (p = 0.011) than that reported in the original work on the development of GASS (14.3±10.5, n = 50; Waddell and Taylor, 2008) [34]. This difference reflects most likely different medications and patients’ characteristics in both studies.

The documentation of the relationship between health literacy and Arabic-GASS score in the present study is of high clinical relevance. In spite of its significance, the influence of health literacy on the perception and reporting of drug side effects is rarely investigated. Extensive literature search allowed us to identify only two studies on this subject. In one, glaucoma patients with limited health literacy were found to be less likely to express problems related to the side effects of their medications to healthcare providers [52]. On the other hand, a second study which included elderly military veterans of the United States did not find a correlation between the health literacy and the reporting of drug side effects [53]. It remains to be determined whether the correlation detected here is specific for patients on antipsychotic drugs, or applies to other groups of patients as well.

Although this is first study to translate GASS to Arabic, it has multiple limitations. First, test-retest reliability was not conducted mainly due to the difficulty in interviewing the same psychitric patients again in a conservative culture where mental illness is regarded as a stigma. Also, this study has a relatively small sample size which was mainly due to the stigma associated with psychiatric illness that made the recruitment of patients very hard [54]. In addition, the Arabic-GASS was not validated against any valid scale in Arabic mainly due to the lack of any validated self-report antipsychotic side effects scale in Arabic. Moreover, this study highlighted on the importance of health literacy in reporting drug side effects among psychiatric patients, however, the used tool was not as reliable as other health literacy screening tools such as the Rapid Estimate of Adult Literacy in Medicine and the Short Test of Functional Health Literacy in Adults [48–50]. Despite these potential imperfections, it is expected that the Arabic-GASS will improve treatment outcomes for Arabic-speaking psychiatric patients by helping them to express their concerns about the side effects of antipsychotic medications. Further, the findings of this study highlight the importance of improving health literacy among psychiatric patients to report the side effects of their medication, which in turn should lead to psotive clinical outcomes. Finally, future studies should translate and validate other self-report antipsychotic side effects scales in Arabic to compare and contrast the performance of Arabic-GASS in detecting antipsychotics’ side effetcs against.

## Supporting information

S1 De-identified data (XLSX)
